# Transmembrane formins as active cargoes of membrane trafficking

**DOI:** 10.1093/jxb/erae078

**Published:** 2024-02-24

**Authors:** Fatima Cvrčková, Rajdeep Ghosh, Helena Kočová

**Affiliations:** Department of Experimental Plant Biology, Faculty of Science, Charles University, Viničná 5, CZ 128 43 Praha 2, Czechia; Department of Experimental Plant Biology, Faculty of Science, Charles University, Viničná 5, CZ 128 43 Praha 2, Czechia; Department of Experimental Plant Biology, Faculty of Science, Charles University, Viničná 5, CZ 128 43 Praha 2, Czechia; University of Glasgow, UK

**Keywords:** Actin, biotic interactions, cell growth, cytokinesis, endocytosis, exocytosis, formin, microtubules, plasmalemma, tonoplast

## Abstract

Formins are a large, evolutionarily old family of cytoskeletal regulators whose roles include actin capping and nucleation, as well as modulation of microtubule dynamics. The plant class I formin clade is characterized by a unique domain organization, as most of its members are transmembrane proteins with possible cell wall-binding motifs exposed to the extracytoplasmic space—a structure that appears to be a synapomorphy of the plant kingdom. While such transmembrane formins are traditionally considered mainly as plasmalemma-localized proteins contributing to the organization of the cell cortex, we review, from a cell biology perspective, the growing evidence that they can also, at least temporarily, reside (and in some cases also function) in endomembranes including secretory and endocytotic pathway compartments, the endoplasmic reticulum, the nuclear envelope, and the tonoplast. Based on this evidence, we propose that class I formins may thus serve as ‘active cargoes’ of membrane trafficking—membrane-embedded proteins that modulate the fate of endo- or exocytotic compartments while being transported by them.

## Teaser

Transmembrane class I formins are cytoskeletal regulators localizing to the plasmalemma and endomembranes that may act as ‘active cargoes’ of membrane trafficking, modulating the fate of membrane compartments carrying them.

## Introduction: formins as and old multifunctional protein family

**Fig. 1.**
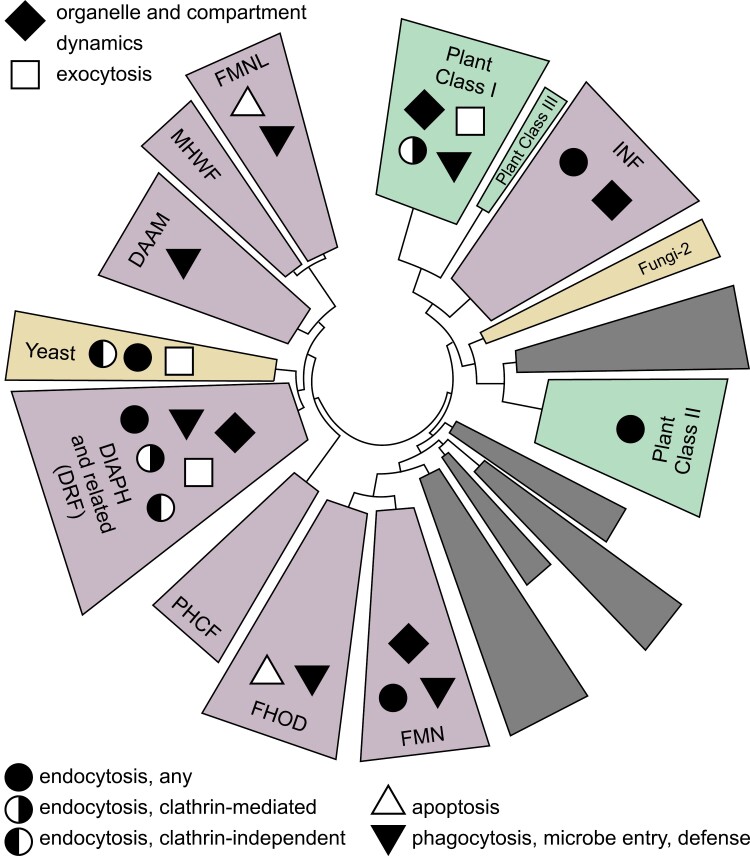
A simplified formin phylogeny with distribution of documented or suspected membrane trafficking-related functions. The schematic FH2 domain tree is based on Pruyne (2017), with only plant, fungal, and major metazoan formin clades labelled. Clades shown in grey correspond to major formin groups found only outside opisthokonts and plants. Branch length is not to scale. Note that the topology of relationships between major clades often lacks statistical support.

The origins of the formin protein family can be traced to the last eukaryotic common ancestor (Grunt *et al.*, 2008; Pruyne, 2017; see also Fig. 1). Defined by the presence of the conserved formin homology 2 (FH2) domain whose dimer can nucleate actin or cap barbed ends of microfilaments (reviewed in Courtemanche, 2018), formins typically contain additional domains and motifs. A proline-rich formin homology 1 (FH1) domain that can recruit profilin-bound actin monomers (Paul and Pollard, 2008; Zweifel and Courtemanche, 2020) is located N-terminally of FH2 in nearly all formins, possibly except some from Rhodophyta (Goodson *et al.*, 2021). Many opisthokont (i.e. fungal, amoebozoan, choanoflagellate, and metazoan) formins, especially members the prototypic Diaphanous-related formin (DRF) clade, contain a conserved GBD/FH3 domain targeted by Rho clade small GTPases, resulting in formin activation through the release of an autoinhibitory interaction between GBD/FH3 and the formin’s C-terminus (reviewed in Kühn and Geyer, 2014; Pruyne, 2017). This regulatory circuit appears to be an opisthokont synapomorphy, since it is present in all examined lineages from metazoans and fungi, as well as in the amoebozoan slime mould *Dictyostelium* and the choanoflagellate *Monosiga*, but absent in other kingdoms including plants (Rivero *et al.*, 2005; Grunt *et al.*, 2008; Pruyne, 2017). Outside of the FH1 and FH2 domains, the structure of formins varies considerably among clades and organismal lineages (e.g. Grunt *et al.*, 2008; Breitsprecher and Goode, 2013; Pruyne, 2016, 2017).

While traditionally understood mainly as actin nucleators, formins are emerging as versatile multifunctional proteins with a variety of cytoplasmic and nucleoplasmic functions. All well-characterized formin roles are so far related either to the actin cytoskeleton (microfilament nucleation, but also capping or bundling; see, for example, Antoku *et al.*, 2023; Ulrichs *et al*., 2023) or to microtubule organization and dynamics. However, since the role of variable domains present in various subsets of formins remains largely uncharacterized, future discovery of cytoskeleton-independent functions cannot be excluded.

Formins can directly bind microtubules and participate in mitotic spindle assembly and function (reviewed in Bartolini and Gundersen, 2010; Mao, 2011; Cvrčková, 2012; Copeland, 2019). Formin–microtubule interactions involve either clade-specific sequence motifs or the FH1 and FH2 domains themselves, or indirect contacts via microtubule-binding proteins (for recent examples from opisthokonts see, for example, Kawabata Galbraith and Kengaku, 2019; Das *et al.*, 2021; and from plants, Sun *et al.*, 2017; Kollárová *et al.*, 2020; Zhang *et al.*, 2021). Interestingly, the actin-sequestering protein profilin, a typical ligand of the FH1 domain, also modulates microtubule dynamics (reviewed in Pinto-Costa and Sousa, 2020), which may account for the engagement of the FH1 domain in some formin–microtubule interactions. However, formin-dependent changes in microtubule dynamics might also be secondary to altered actin organization.

The metazoan formin INF2 may indirectly control tubulin acetyltransferase expression, and hence tubulin acetylation, via depletion of actin monomers whose level is sensed by certain transcription factors (see Fernández-Barrero and Alonso, 2018). INF2 also controls nuclear actin assembly on the inner face of the nuclear membrane (Wang *et al.*, 2019). Another metazoan formin, DAAM, participates in nuclear actin-mediated control of transcription as well (Knerr *et al.*, 2023), documenting the contribution of formins to the control of gene expression (compare Isogai and Innocenti, 2016). A recent report (K. Zhang *et al.*, 2023) identifies a mammalian DRF family member as a central component of stress-induced protein aggregates, suggesting regulation of its actin-organizing activity by sequestration due to liquid–liquid phase separation.

In the plant kingdom, research has focused mainly on two well-established formin clades present in seed plants, termed class I and class II (Deeks *et al.*, 2002; Grunt *et al.*, 2008), while a third clade (class III), documented in algae, mosses, and lycophytes, but absent in angiosperms (Grunt *et al.*, 2008), remains largely unexplored. Here we examine evidence suggesting the participation of plant formins in membrane trafficking, building both on plant data and on observations from other biological systems. We specifically focus on angiosperm class I formins—a unique group of formins that are typically also integral membrane proteins (Cvrčková, 2000) and thus have to pass through compartments of the endomembrane system before reaching their final cellular destination.

## Membrane-associated formins across kingdoms

While all non-plant formins studied to date, as well as most plant formins, are cytoplasmic or nuclear, they nevertheless commonly associate with membranes. A phosphoinositide-binding domain related to the PTEN (phosphatase and tensin homologue) protooncogene, first discovered in plant class II formins, is also present in formins of choanoflagellates and some fungi, suggesting that ancestral formins contained a similar domain mediating peripheral membrane attachment (Pruyne, 2017). In *Physcomitrium patens*, the PTEN-related domain of class II formin For2a binds phosphoinositide-3,5-bisphosphate [PtdIns(3,5)P_2_] and localizes the formin to PtdIns(3,5)P_2_-rich plasmalemma regions (van Gisbergen *et al.*, 2012).

Many formins interact with small GTPases, which themselves are peripheral membrane proteins. This could confer membrane localization to cytoplasmic formins, as proposed for metazoan DRFs containing a typical Rho GTPase-binding GBD/FH3 motif. However. GBD/FH3 alone is not sufficient for membrane localization of mouse formin mDia2 (Gorelik *et al.*, 2011). An mDia2-derived peptide outside GBD/FH3 is predicted to bind membranes enriched in PtdIns(4,5)P_2_ in molecular dynamics simulations, and *in vitro* actin nucleation on PtdIns(4,5)P_2_-enriched membranes appears to require formin activity (Bucki *et al.*, 2019). However, the latter observation builds on pharmacological experiments utilizing the broad-spectrum formin inhibitor SMIFH2 (small molecule inhibitor of FH2) that might affect additional targets including myosins (Nishimura *et al.*, 2021; Innocenti, 2023). Formin localization at the cytoplasmic face of the plasmalemma is also controlled by membrane phosphoinositide content in crawling *Dictyostelium* amoebae, where distinct DRF isoforms participate in the formation of actin wave patterns (Ecke *et al.*, 2020).

While plants do not have DRFs, algal, moss, or lycophyte class III formins typically contain a different Rho GTPase-binding domain, related to a Rho GTPase-activating protein (Grunt *et al.*, 2008). To date, we are not aware of functional studies addressing this formin clade besides one report documenting low to undetectable levels of *P. patens* For3 expression in vegetative tissues, with no localization, biochemical, or genetic data (Vidali *et al.*, 2009).

Peripheral formin attachment to membranes may be mediated by other interacting proteins, such as transmembrane receptors, BAR domain- or FYVE domain-containing proteins, as documented in metazoans. Some of them also have homologs in plants (reviewed in Cvrčková, 2013). For the Arabidopsis BAR domain-containing protein SH3P3, binding to the cytoplasmic part of the transmembrane class I formin AtFH5 was documented (Baquero Forero and Cvrčková, 2019). Although the biological significance of this finding remains unclear, it shows that formin–BAR domain interaction can occur in plants (though in this case not as a mechanism of formin localization).

Plant class I formins present an exception from the near-universal cytoplasmic (or nuclear) formin localization, being typically (though not always) integral membrane proteins with an N-terminal secretory signal and one transmembrane helix. They are predicted to insert co-translationally into membranes, with the FH1 and FH2 domains located in the cytoplasm (Cvrčková, 2000; Grunt *et al.*, 2008; Pruyne, 2017), and the extracytoplasmic portion, often proline rich, exposed to the extracytoplasmic space. Reminiscent of metazoan integrins, they could link the plasmalemma, cortical cytoskeleton, and the cell wall, possibly engaging in mechanotransduction (see Ma *et al.*, 2023). Using fluorescence recovery after photobleaching with fluorescent protein-labelled formin derivatives, Martiniere *et al.* (2011) documented that the extracytoplasmic part of the Arabidopsis class I formin AtFH1 binds to the cell wall. Interestingly, while opisthokont formins typically are processive (i.e. remain attached to the barbed end of microfilaments; see, for example, Mizuno *et al.*, 2018), AtFH1 detaches from actin filaments soon after nucleation, thus providing the first example of a non-processive formin (Michelot *et al.*, 2006; reviewed in Blanchoin and Staiger, 2010). This may provide a means for relieving the supercoiling that would otherwise result if a cell wall-anchored formin, unable to rotate, remained attached to the barbed end of microfilaments while adding new actin monomers.

The characteristic domain layout of plant class I formins appears to be unique to the plant kingdom (Box 1). Nevertheless, given the numerous cases of peripheral membrane association and membrane-related functions of opisthokont cytoplasmic formins, observations from non-plant organisms may be relevant for understanding the roles of formins in the endomembrane system.

Box 1. **Transmembrane formins as a probable plant synapomorphy**

We reported four theoretically predicted putative non-plant integral membrane formins in our previous phylogenetic study, built in part on pre-annotation versions of genome sequences (Grunt *et al.*, 2008), one from the annelid worm *Helobdella robusta*, one from *Caenorhabditis elegans*, and two from the amoeba *Naegleria gruberi*. However, the corresponding database records have since been updated, and none of their current GenBank versions (accession nos XP_009023422.1, NP_503132.3, XP_002682144.1, and XP_002679869.1) carries a putative signal peptide, although some do contain predicted transmembrane helices. Thus, there is currently no evidence supporting the existence of non-plant integral membrane formins. Transmembrane formins appear to be unique to the plant kingdom, and genes encoding them are found in every plant genome examined so far, suggesting that this formin organization is most probably a plant synapomorphy.

## Formins in membrane trafficking: lessons from opisthokonts

Eukaryotic membrane trafficking is intimately linked with actin dynamics at many steps, from endocytotic vesicle formation, through myosin-driven vesicle transport, to vesicle delivery to endomembrane or plasmalemma destinations (reviewed, for example, in Chakrabarti *et al.*, 2021). All these processes can be modulated to a greater or lesser extent by formins via their actin-mediated effects. We will focus on cases where either a formin was found to localize directly to compartments of the endomembrane system, or a specific formin role in endocytosis, exocytosis, or intercompartment membrane trafficking has been documented or proposed. While these roles are usually actin mediated, the general contribution of actin to membrane trafficking is beyond the scope of this review.

Remarkably, even considering the (major) differences in experimental coverage of various formin clades, endomembrane-related functions of opisthokont formins appear to be distributed across the whole phylogenetic diversity of the formin family (Fig. 1). These formin roles are thus likely to be ancestral and relevant for all lineages including plants.

### Formins in endocytosis, endosomal trafficking, and autophagy

Although participation of formins in endocytotic membrane trafficking has been studied for more than two decades, we are not aware of any comprehensive review of this subject. We thus summarize the key relevant findings below, including older studies.

The budding yeast (*Saccharomyces cerevisiae*) formin Bni1 is an essential component of a clathrin-independent endocytotic pathway (Prosser *et al.*, 2011; Woodard *et al.*, 2023). In the fission yeast (*Schizosaccharomyces pombe*), formins are required for endocytosis spatially restricted to certain regions of the cell surface (Gachet and Hyams, 2005; Onwubiko *et al.*, 2019). In various metazoan cell types, including epithelia and neurons, DRF clade formins participate in both clathrin-independent and clathrin-mediated endocytosis (Gasman *et al.*, 2003; Levayer *et al*., 2011; Soykan *et al.*, 2017). Moreover, Rho GTPase-controlled, DRF-mediated actin assembly on endosomes is essential for their trafficking in various mammalian cell types (Fernandez-Borja *et al.*, 2005; Wallar *et al.*, 2007).

While most studies linking metazoan formins to endocytosis involve members of the DRF clade, other clades also have endocytosis-related roles. Mouse Fmn2 is required for neural progenitor proliferation due to its participation in membrane receptor endocytosis and subsequent lysosomal degradation (Lian *et al.*, 2016, 2019), while the endoplasmic reticulum- and plasmalemma-localized INF2 formin controls the amount of the CFTR (cystic fibrosis transmembrane conductance regulator) chloride channel at the plasmalemma in a manner consistent with its role in CTFR internalization (Santos *et al.*, 2020). A role in receptor internalization was also reported for the DRF formin Diaph1/mDia1 in hepatic stellate cells (Liu *et al.*, 2020).

An SH3 domain-containing formin- and dynamin-binding protein, FBP17 or Rapostlin, contributes to the formation of endocytotic plasma membrane invaginations in several mammalian cell types including neurons (Kamioka *et al.*, 2004; Wakita *et al.*, 2011), although participation of any formin in this process is yet to be demonstrated. Another mammalian formin interactor, FNBPL1, is essential for autophagy (Huett *et al.*, 2009), suggesting a wider role for formins in endomembrane trafficking. Moreover, *Drosophila* genetic screens identified formins of FHOD and FMNL clades as components of apoptotic signalling and execution pathways, documenting their participation in processes involving major endomembrane system rearrangements (Anhezini *et al.*, 2012; Fullard and Baker, 2015). The function of formins themselves may also be modulated by endocytosis; in a *Drosophila* embryo wound healing model, endocytosis is required for proper coordination of (partly) DRF-dependent actin assembly (Matsubayashi *et al.*, 2015).

Formins also contribute to phagocytosis, not only in various metazoan cell types and diverse contexts (Colucci-Guyon *et al.*, 2005; Brandt *et al.*, 2007; Lewkowicz *et al.*, 2008; Junemann *et al.*, 2016), but also in *Entamoeba* (Bharadwaj *et al*., 2018, 2021) and *Dictyostelium* (Körber *et al*., 2023). Formin-dependent phagosome formation may facilitate microbial pathogen internalization by host cells or spreading across tissues (Naj *et al.*, 2013; Hoffman *et al.* 2014; Rengarajan *et al.*, 2016; Williams *et al.*, 2016; Dhanda *et al*., 2021). While this may not seem directly relevant for plants, which do not perform phagocytosis in the classical sense, some plant–microbe interactions, such as entry of rhizobia into root hairs, may involve phagocytosis-like mechanisms (Yutin *et al.*, 2009), and formins do play a part in the contacts between plant cells and symbiotic or pathogenic microorganisms (see below).

### Formins in the secretory pathway and associated with organelles

Several recent reports indicate formin participation in exocytotic trafficking, involving both DRFs and members of other formin clades. In mouse mast cells, the DRF formin mDia1 promotes cell migration and inhibits secretion via actin cytoskeleton rearrangements (Klein *et al.*, 2019). Another DRF, human DIAPH1, is required for secretory cargo trafficking to the base of primary cilia of immortalized retinal pigmental epithelial cells (Palander and Trimble, 2020). Formin-controlled actin rearrangements also drive secretory vesicle fusion in bovine chromaffin cells (Wen *et al.*, 2016). However, since this conclusion is based on experiments utilizing SMIFH2, the above-discussed specificity concerns remain relevant (see Innocenti, 2023).

Formins also contribute to relocation of larger compartments of the endomembrane system, as well as endosymbiotic organelles. Myosin V-dependent movement of mammalian melanosomes—membrane-bounded compartments harbouring constituents and products of the melanin biosynthesis pathway—is regulated by effectors of the small GTPase Rab 27a that include the actin nucleator SPIRE and the formin FMN1, engaged in the assembly of actin tracks for the movement of these organelles (Alzahofi *et al.*, 2020). The motility of mitochondria in both mammalian and *Drosophila* cells depends on DRF-clade formins by a mechanism involving actin polymerization (Minin *et al.*, 2006; Li and Sewer, 2010). Fission of mammalian mitochondria requires the activity of INF clade formins to generate actin assemblies necessary for mitochondrial constriction (see Tilokani *et al.*, 2018; Fung *et al.*, 2023) and, according to a recent report (Gatti *et al.,* 2023, Preprint), INF2-dependent actin polymerization is also required for mitochondrial fusion. Moreover, the C-terminal autoinhibitory domain of mouse DRF Diaphanous-1 was found to bind the mitofusin MFN2, a protein engaged in mitochondrial fusion and in tethering of mitochondria to the endoplasmic reticulum (Yepuri *et al*., 2023).

Participation in membrane fusion was also documented for the fission yeast formin Fus1, which focuses actin- and myosin V-driven exocytosis during gamete fusion. Remarkably, formation of molecular condensates involving an intrinsically disordered part of the formin molecule is essential for this process (Billault-Chaumartin *et al.*, 2022; Jacobs *et al.*, 2022). In the budding yeast and filamentous fungi, formins act as core components of the polarisome (see Xie and Miao, 2021)—a large signalling and structural complex governing tip growth, a process that has its counterpart in plant cells, albeit without the participation of a polarisome-like structure (recently reviewed, for example, in R. Zhang *et al.*, 2023).

## Plant class I formins at the expanding plasmalemma

**Fig. 2.**
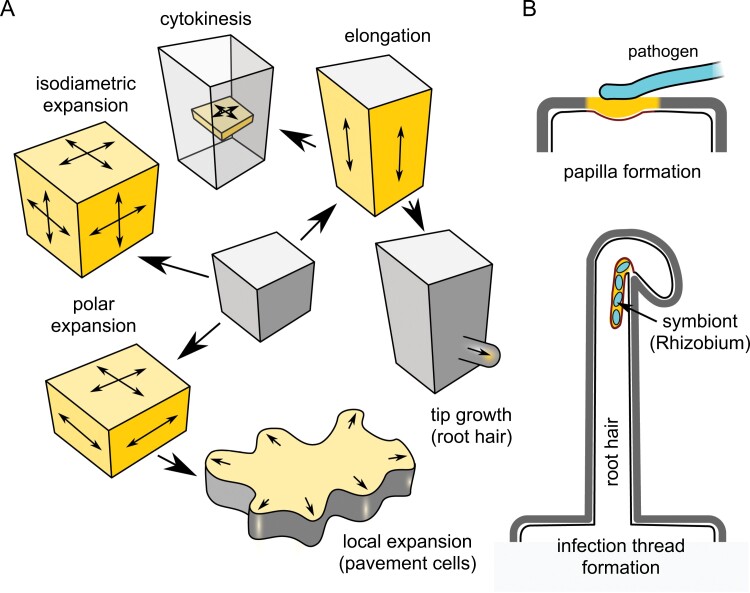
Modes of plasmalemma expansion and targeted secretion discussed in this review. (A) Plasmalemma expansion during cell growth and division. (B) Targeted secretion during biotic interactions. Destinations of exocytosis, coinciding with areas of vigorous plasmalemma turnover that correspond to activated cortical domains *sensu*Žárský *et al.* (2009), are highlighted. (B) is in part based on Rae *et al*. (2021).

Plant cell growth depends on the delivery of membrane-bounded vesicles carrying membrane lipids and proteins, but also exocytotic cargoes contained within vesicles, to the plasmalemma. The diversity of possible spatial configurations of plasma membrane expansion (Fig. 2) might be one of the factors contributing to the large number of paralogues of genes engaged in these processes. The amount of exocytosed membrane usually exceeds the plasmalemma area increase necessary for cell growth, and control of cell shape often involves signalling modulated by membrane receptors that undergo turnover. Thus, endocytotic membrane retrieval must take place in growing cells. Exo- and endocytosis have to be precisely coordinated (see, for example, Orr *et al.*, 2020), and, similar to the opisthokonts, in plants many steps in these processes also rely on the function of the actin cytoskeleton (see, for example, Wang and Hussey, 2015).

**Fig. 3.**
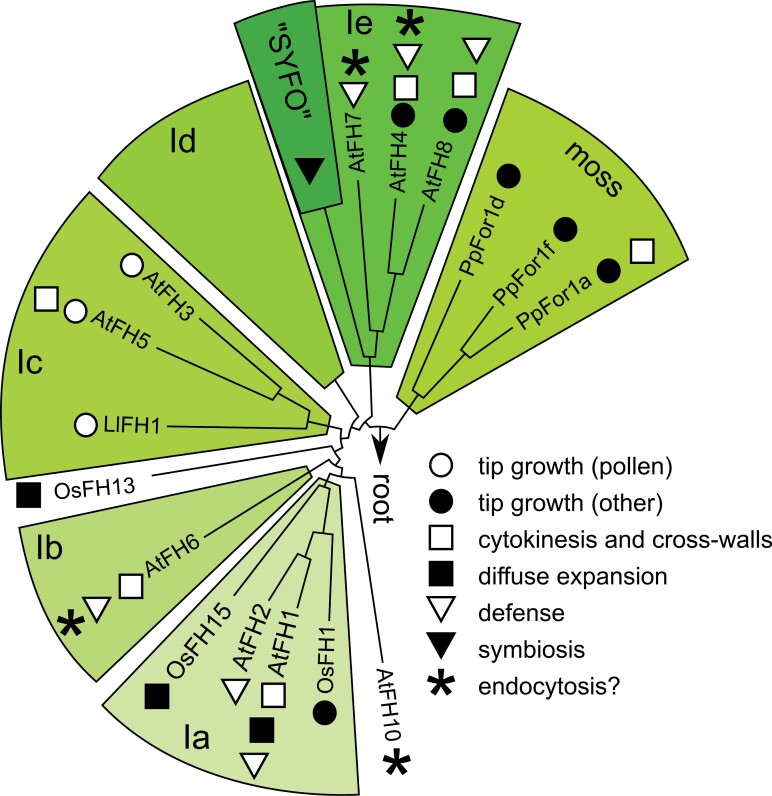
Phylogenetic distribution of cell expansion- and membrane trafficking-related roles of class I formins. The schematic FH2 domain tree is based on previous reports (Cvrčková *et al*., 2004; Li *et al*. 2017; van Gisbergen *et al*., 2018; Liang *et al*. 2021). Highlighted branches correspond to evolutionarily conserved clades within the class I formin family; only formins discussed in this review are shown individually. Branch length is not to scale.

Transmembrane formins could participate in plasmalemma expansion by affecting not only cytoskeletal organization, but also endo- and exocytosis. The multiple class I formin paralogues are likely to have overlapping functions, and mutant phenotypes are thus not expected to be dramatic, which indeed appears to be the case. Despite this ‘functional redundancy’, distinct evolutionarily conserved subclades of the class I formin family (see Cvrčková *et al.*, 2004; Liang *et al.*, 2021) may be preferentially associated with specific modes of local plasmalemma expansion or specific plasmalemma-targeted traffic (Fig. 3).

### Tip growth

While superficially similar in their cell expansion mode, the two best characterized tip-growing seed plant cell types—pollen tubes and root hairs—exhibit distinct actin arrangements specific for one or the other (e.g. Ketelaar 2013; Xu and Huang, 2020), with a characteristic ‘actin fringe’, which may act through focusing exocytosis to the expanding apical dome, present in the former but not in the latter beyond the initial bulge stage (reviewed in Stephan, 2017). We cannot therefore assume a similar role for formins in pollen tubes and in elongating root hairs, were they contributing only through modifying actin organization.

The first observation of a class I formin acting in tip growth comes from heterologous ectopic overexpression of Arabidopsis AtFH1, normally expressed in vegetative tissues but barely detectable in the male gametophyte, in tobacco pollen. In this setup, AtFH1 caused bulging of pollen tube tips, excessive microfilament bundling, plasmalemma invaginations, and defective actin fringe formation (Cheung and Wu, 2004). Similar defects were elicited by overexpression of a deletion derivative of the predominant Arabidopsis class I formin of the male gametophyte, AtFH3, while RNAi-mediated suppression of AtFH3 expression inhibited pollen tube growth (Ye *et al.*, 2009). Loss of function of the closely related and also pollen-expressed AtFH5, as well as a double *atfh3 atfh5* mutation, disrupts actin fringe formation and results in wavy, somewhat thickened pollen tubes (Cheung *et al.*, 2010; Lan *et al.*, 2018). While both AtFH3 and AtFH5 localize to the plasma membrane and endomembranes of the pollen tube tip, participate in actin nucleation at the plasmalemma, and contribute to actin fringe organization, they exhibit distinct plasmalemma localization patterns; AtFH5 is restricted to the tip, while AtFH3 also decorates the tube shank (Lan *et al.*, 2018). This difference appears to be due to distinct glycosylation patterns of the extracytoplasmic part of these formins and restriction of their lateral mobility by binding to specific cell wall components, as documented by effects of mutations affecting the glycosylation pathway or target glycosylation sites on localization of these formins (Lara-Mondragón *et al.*, 2022). AtFH5 localizes to exocytotic vesicles before pollen tube emergence, participating directly in profilin-aided actin nucleation on them (C. Liu *et al.*, 2018, 2021). Periodic delivery of AtFH5-decorated vesicles carrying the calcium-permeable channel CNGC18 to the pollen grain germination site has been recently shown to contribute to oscillatory changes in cytoplasmic calcium that contribute to step-wise bulge expansion at the onset of pollen tube germination (Ruan *et al.*, 2023). A lily formin closely related to AtFH3 and AtFH5, LlFH1, which can nucleate, cap, and bundle microfilaments *in vitro*, also localizes to exocytotic vesicles in the actin fringe area and modulates pollen tube elongation through coordinating actin organization with membrane trafficking, as documented by altered actin fringe organization and slower elongation of pollen tubes with altered expression of this formin (Li *et al.*, 2017).

Regulation of several formins, including possibly class I representatives, by miRNAs derived from hairpin segments of long single-stranded transcripts was recently suggested to contribute to male sterility in wheat, implying a possible impact on pollen tube growth (Duan *et al.*, 2021). It is not clear whether the targeted formins were class I members due to a methodological flaw in the presented phylogenetic analysis, which included not only domains present in all formins analysed (i.e. FH2) but also variable domains without shared evolutionary history (Duan *et al.*, 2021). Nevertheless, at least one Arabidopsis class I formin, AtFH1, appears as a possible target of RNA-mediated post-transcriptional regulation that may be worth investigating in the endomembrane context (Box 2).

Box 2. **Possible RNA-mediated regulation of plant formin expression?**

Our bioinformatic analyses uncovered a putative natural antisense transcript [a possible long non-coding RNA (lncRNA) precursor] targeting the main *A. thaliana* housekeeping class I formin, AtFH1 (At3g25500), overlapping with the 3'-untranslated region of the adjacent protein kinase-encoding locus At3g25490. In the newest *A. thaliana* genome annotation (Cheng *et al.*, 2017), this locus is described as a putative formin fragment-encoding locus (At3g25493); however, there is currently no evidence for its transcription in either the sense or antisense orientation. The putative antisense transcript is well conserved in genomes of multiple *A. thaliana* accessions from GenBank or the 1001 genomes database (1001 Genomes Consortium, 2016) but absent in closely related *A. lyrata*. The locus is well conserved among Col-0 and the additional nine accessions examined (Ler-0, Kn-0, Cdm-0, Ty-1, Eri-1, Sha, Cvi-0, An-1, and Kyo), while a further four (Ga-0, Mitterberg-2-185, Mitterberg-3-189, and Olympia-2) have a short deletion in a part of the predicted transcript upstream of the area matching the AtFH1 locus. No other class I formin-encoding Arabidopsis loci appear to be targeted by similar well-conserved transcripts, although a putative lncRNA located next to the class II formin-encoding locus AtFH17 (At3g32400), possibly targeting one or more class II formin genes, has also been found. In addition, a putative circular non-coding RNA targeting AtFH14, another class II formin locus, has been reported (Frydrych Capelari *et al*., 2019). These findings deserve further investigation also in the endomembrane context, since recognition and degradation of target mRNAs may take place on the endoplasmic reticulum and other membrane compartments (see Kim *et al.*, 2014), and could thus be affected by changes in membrane trafficking. Moreover, endocytosis in some opisthokont systems is controlled by miRNAs targeting, among other proteins, Rab GTPases and dynamins (Serva *et al.*, 2012; Aranda *et al.*, 2015). The repertoire of plant dsRNAs and lncRNAs indeed changes dramatically in situations involving reorganization of the membrane trafficking pathways, such as, for example, during pathogen response (Nie *et al.*, 2021).

The role of class I formins in root hair development has received, so far, much less attention than that in pollen tubes. Overexpression of two closely related Arabidopsis formins, AtFH4 and AtFH8, caused root hair branching (i.e. ectopic tip formation), while a dominant negative AtFH8 allele inhibited root hair initiation and growth (Deeks *et al.*, 2005; Yi *et al.*, 2005). Mutants with impaired function of rice OsFH1 also exhibit reduced root hair growth (Huang *et al.*, 2013). According to pharmacological studies employing SMIFH2, formin function is also required for nuclear migration in root hairs (Zhang *et al.*, 2019), a process of unclear relevance for tip growth.

Besides pollen tubes and root hairs, tip growth, or at least a related cell expansion mode, may occur in other cell types. Unicellular trichomes are a prime example, studied especially in the commercially important model of cotton fibres, whose cytoskeletal organization exhibits some features of both diffuse and tip growth modes (Yu *et al.*, 2019). Genes presumably encoding class I formins (annotated as close homologues of AtFH1 and AtFH5) are differentially regulated in relevant ovule and seed coat tissues during early stages of cotton fibre formation (Qin *et al.*, 2022). In Arabidopsis, AtFH1 localizes to nascent trichome tips at early developmental stages; somewhat surprisingly, its overexpression may result in misshapen trichomes, sometimes with a reduced number of branches (Oulehlová *et al.*, 2019), while its loss increases trichome branch numbers and trichome shape complexity (Cifrová *et al.*, 2020). While this might suggest a possible regulatory role for this formin in shaping the developing trichomes, the phenotype could also be explained as resulting from increased overall microtubule dynamics in *atfh1* mutants, which might generally facilitate morphogenetic processes in this cell type, as well as in others, especially epidermal pavement cells (Rosero *et al*., 2016; see also below).

Outside vascular plants, tip growth is well characterized in moss protonemata, building mainly on the *P. patens* model, where distinct class I formin paralogues, located to the plasmalemma and cortical exocytotic foci, differentially contribute to the organization of specific actin arrays during tip growth (van Gisbergen *et al.*, 2020). Remarkably, mosses contain a unique group of class I formin paralogues, represented by the *P. patens* essential gene For1F, where a Sec10-like domain replaces the usual membrane localization motifs, linking the characteristic FH1 and FH2 domains of a formin to the hetero-octameric exocyst protein complex engaged in membrane vesicle targeting. Loss-of-function mutation can be rescued by overexpression of either the formin or Sec10-like parts of the protein, and even of another Sec10 paralogue, suggesting that physical fusion of the two domains can be replaced by transient interactions of proteins expressed to a high level, and that the vital role of For1F involves exocytosis rather than only actin organization (van Gisbergen *et al.*, 2018).

Participation of plant formins in tip growth is not limited to the class I clade. Loss-of-function mutants in the rice class II formin FH5, also known as Bent Upper Internode (BUI) or Rice Morphology Determinant (RMD), exhibit decreased pollen germination and tube growth rates, as well as deformed pollen tubes (Li *et al.*, 2018). In Arabidopsis, loss of the pollen-expressed class II formin AtFH13, which localizes to intracytoplasmic (endomembrane?) structures, surprisingly stimulates pollen tube elongation, while its overexpression inhibits pollen tube growth (Kollárová *et al.*, 2021). In *P. patens*, the class II formin For2a localizes to endocytotic foci at the apices of tip-growing protonemata, where it drives the assembly of actin arrays used for myosin-dependent membrane vesicle delivery (Vidali *et al.*, 2009; van Gisbergen *et al.*, 2012, 2020; Galotto *et al.*, 2021). This suggests a possible ‘division of labour’ between moss class I and class II formins in tip growth, with the former mainly contributing to exocytosis and the latter to endocytosis. Given the specific features of the moss system, generalization towards angiosperms would be merely speculative (although possibly consistent with the above-mentioned *atfh13* mutant and overexpression phenotypes, but not with the rice *rmd* mutant phenotype).

### Cytokinesis and diffuse cell expansion

Cell plate assembly during cytokinesis could be understood as the deposition of new membrane and primary cell wall material to a (transiently) intracellular compartment rather than the cell surface (see Fig. 2A). Application of the formin inhibitor SMIFH2 in tobacco cell cultures disrupts cytokinesis by disturbing predominantly microtubule, but also actin and dynamin, function (Zhang *et al.*, 2021). Several Arabidopsis class I formins localize to the nascent cell plate, including AtFH5, whose mutation delays endosperm cellularization (Ingouff *et al.*, 2005), as well as AtFH1 (Oulehlová *et al.*, 2019) and AtFH8 (Xue *et al.*, 2011; Zhang *et al.*, 2021). Mutations affecting either AtFH1 or AtFH8 did not produce a noticeable cytokinesis-related phenotype, probably due to functional redundancy. In *P. patens*, the class I formin For1A but not its relative For1D is found at the cell plate (van Gisbergen *et al.*, 2020), indicating subfunctionalization among formin paralogues. While class II formins also participate in cytokinesis, Arabidopsis AtFH14, whose suppression by RNAi disrupts microspore division, localizes to the phragmoplast rather than the cell plate (Li *et al*., 2010).

Following cytokinesis, class I formins may stay, at least transiently, at the plasmalemma, sometimes remaining focused to cross-walls of cell files within the root, as shown, for example, for AtFH4 and AtFH8 (Deeks *et al.*, 2005) or AtFH6 (Van Damme *et al.*, 2004). The main Arabidopsis housekeeping formin AtFH1, expressed (to somewhat varying levels) in most vegetative tissues with a maximum in the root tip, subsequently relocates to plasmodesmata and endosomes, with a substantial plasmalemma-located fraction remaining in the root meristematic and transition zones (Oulehlová *et al.*, 2019). When overexpressed, AtFH1 remains at the plasma membrane in other tissues as well (Martinière *et al.*, 2011). Minor quantitative alterations in rhizodermis cell expansion and cotyledon tissue growth were observed in mutants with impaired function of AtFH1 upon treatment with low doses of anti-actin and anti-microtubule drugs, indicating an increased sensitivity to both microfilament and microtubule perturbation (Rosero *et al.*, 2013). Subsequent *in vivo* actin and microtubule dynamics observations suggest participation of AtFH1 in actin–microtubule crosstalk (Rosero *et al.*, 2013, 2016). Mutant cotyledons also exhibit noticeably more complex epidermal pavement cell shape (Rosero *et al.*, 2016; Cifrová *et al.*, 2020); that is, an alteration in local cell expansion that depends on orchestrated action of the cytoskeleton and membrane trafficking, including also that of auxin carriers (see S. Liu *et al*., 2021). Remarkably, while some signalling proteins engaged in pavement shape control, such as, for example, the pleckstrin homology ROP GTPase-activating proteins (PHGAPs), preferentially localize to nascent pavement cell indentations (C. Zhang. *et al.*, 2022)—to the sites where the first observable cytoskeletal rearrangements take place during the establishment of lobe layout (Armour *et al.*, 2015), no such local enrichment at either lobes or indentations was observed for AtFH1, which instead concentrates at plasmodesmata in epidermal pavement cells, together with some other class I formins (Diao *et al.*, 2018; Oulehlová *et al.*, 2019).

The effects of *atfh1* mutations on pavement cell shaping can be interpreted as consequences of changes in actin and especially microtubule dynamics and arrangement rather than membrane trafficking (see also Cvrčková *et al.*, 2016). The decreased hypocotyl elongation rate in young *atfh1* seedlings has also been attributed to changes in actin organization (Cui *et al.*, 2023). In developing rice grains, loss of function or RNAi suppression of the class I formin OsFH15 led to decreased cell expansion in the spikelet hull tissues, resulting in smaller grain size, while OsFH15 overexpression facilitated spikelet hull tissue expansion, leading to larger grains. These changes were also attributed to altered cytoskeletal organization rather than membrane trafficking (Sun *et al.*, 2017).

Formins might thus contribute to diffuse cell expansion mainly through their effects on the cytoskeleton, and observed changes in membrane trafficking may be secondary to cytoskeletal alterations. This applies not only to class I formins, but also to their class II paralogues. An especially intricate connection to endomembrane dynamics has been described for the rice RMD/BUI/FH5 class II formin, whose pleiotropic mutant phenotype involves diffuse cell expansion defects that can be due to a combination of cytoskeletal changes and altered recycling of PIN auxin transporters. At the same time, the transcription of RMD is controlled by an auxin-responsive transcription factor. This results in a feedback loop involving auxin-controlled transcription of a cytoskeletal organizer that modulates trafficking of auxin transporters and subsequently polar auxin transport (Li *et al.*, 2014).

## Class I formins in biotic interactions: beyond defence?

The plasmalemma, together with the cell wall, is the first site of contact between the plant and interacting (micro)organisms, be they pathogens, parasites, or beneficial symbionts. On the plant side, these interactions involve major changes in (mainly secretory) membrane trafficking (reviewed in Yun *et al.*, 2023). Formins may contribute to these processes in several situations.

The first sign of engagement of class I formins in biotic interactions came from the finding that Arabidopsis AtFH6 decorates the plasmalemma of nematode-induced giant cells and its expression increases upon nematode infestation, although its loss did not noticeably change the plants’ response to the parasite, possibly due to functional overlap among formins (Favery *et al.*, 2004). AtFH6 undergoes clustering at the plasmalemma, modulated by microbe-derived signalling molecules such as the pathogen-associated molecular pattern (PAMP) flagellin or the bacterial quorum-sensing diffusible signal factor, resulting in changes in actin nucleation at the membrane (Ma *et al.*, 2021). Enhanced actin nucleation upon PAMP induction is aided by the interaction between AtFH6 and the membrane protein remorin, resulting in membrane nanodomain reorganization (Ma *et al.*, 2022). Formin-controlled actin dynamics during pathogen response may be further modulated by PAMP-triggered oligomerization of some profilin isoforms, turning profilin into an inhibitor rather than a cofactor of formin-driven actin polymerization (Sun *et al.*, 2018).

Plant cells often use localized secretory deposition of cell wall appositions or defensive papillae to prevent entry of microbial pathogens, especially fungal hyphae (see Fig. 2B). The Arabidopsis class I formin AtFH4, whose transcription is induced by many types of biotic stress, accumulates at mobile endomembrane compartments adjacent to sites of fungal penetration attempts, and drives a localized defensive secretion of a specific set of cargoes; simultaneous mutation of AtFH4 and its closest paralogues AtFH7 and AtFH8 increases sensitivity towards fungal pathogens (Sassmann *et al.*, 2018). The housekeeping formin AtFH1, together with its close relative AtFH2, also contributes to defensive papilla formation by participating, together with the Arp2/3 complex, in the organization of actin patches driving defensive secretion and membrane recycling (Qin *et al.*, 2021).

Besides defence against pathogens, formins contribute to symbiotic plant–microbe interactions. Members of the *Medicago truncatula* SYFO (Symbiotic formin) class I subclade, closely related to Arabidopsis AtFH4, AtFH7, and AtFH8, participate in cytoskeleton and endomembrane rearrangements in root hairs accommodating *Rhizobium* symbionts, including formation of plasmalemma invaginations in response to rhizobial nodulation factors (Liang *et al.*, 2021; compare Fig. 2B).

Known roles of formins in plant biotic interactions thus so far appear to be restricted to class I members (especially from the SYFO clade) and to the exocytotic pathway; however, mapping of this area of formin functioning is still in its infancy.

## Class I formins in the endomembrane system

The above-described cases of class I formins documented or suspected to participate in membrane turnover are summarized in Table 1. However, there are also additional instances of endomembrane localization of class I formins whose biological implications are unknown (i.e. not necessarily related to membrane trafficking).

**Table 1.** Summary of documented class I formin activities and localizations related to membranes and membrane trafficking

Localization	Species	Formin	Description	Reference
Plasmalemma	*A. thaliana*	AtFH1	Localizes to PM of pollen tube tips when heterologously overexpressed. At cell plate and PM in root meristem and in nascent trichome tips. Lateral mobility at the PM is restricted by cell wall interactions.	Cheung and Wu (2004); Martinière *et al.* (2011); Oulehlová *et al*. (2019)
	*A. thaliana*	AtFH3	Localizes to a distinct domain of pollen tube tip PM, different from AtFH5.	Lan *et al.* (2018)
	*A. thaliana*	AtFH4	Locates to the PM at contacts of sister cells in the rhizodermis.	Deeks *et al*. (2005)
	*A. thaliana*	AtFH5	Localizes to a distinct domain of pollen tube tip PM, different from AtFH3, and to the nascent cell plate.	Ingouff *et al.* (2005); Lan *et al.* (2018)
	*A. thaliana*	AtFH6	Found at PM of nematode-induced giant cells.	Favery *et al.* (2004)
	*A. thaliana*	AtFH8	Locates to the cell plate and to PM at contacts of sister cells in the rhizodermis.	Deeks *et al*. (2005); Xue *et al.* (2011); Zhang *et al.* (2021)
	*O. sativa*	OsFH1	Found at the PM.	Huang *et al.* (2013)
	*O. sativa*	OsFH13	At PM, relocates to chloroplast envelope upon light stimulus.	Y. Zhang *et al.* (2023)
	*P. patens*	For1A	At cell plate and PM, localization differs from For1D	van Gisbergen *et al*. (2020)
	*P. patens*	For1D	At the PM, localization differs from For1A	van Gisbergen *et al*. (2020)
	*P. patens*	For1F	At exocytotic foci of the PM (a moss-specific Sec10–formin fusion protein)	van Gisbergen *et al*. (2018)
Endoplasmic reticulum	*A. thaliana*	AtFH4	Overexpression of tagged protein aligns the ER to microtubules.	Deeks *et al*. (2010)
Nuclear envelope	*A. thaliana*	AtFH5	A truncated derivative localizes to the nuclear envelope.	Cvrčková *et al.* (2014)
	*A. thaliana*	AtFH8	At the nuclear envelope in interphase meristematic cells.	Xue *et al.* (2011)
Tonoplast	*A. thaliana*	AtFH1	Transiently decorates the tonoplast in the root transition zone.	Oulehlová *et al*. (2019)
Organelles	*O. sativa*	OsFH13	Localizes to chloroplast envelope upon light stimulus.	Y. Zhang *et al.* (2023)
Exocytotic vesicles	*A. thaliana*	AtFH5	Nucleates actin on exocytotic vesicles.	C. Liu *et al.* (2018, 2021)
	*L. longiflorum*	LlFH1	Nucleates actin on exocytotic vesicles.	Li *et al.*, 2017
Endocytotic pathway compartments	*A. thaliana*	AtFH1	Found at early endosomes.	Oulehlová *et al*. (2019)
	*A. thaliana*	AtFH4	Found in the proteome of clathrin-coated vesicles.	Dahhan *et al.* (2022)
	*A. thaliana*	AtFH6	Found in the proteome of clathrin-coated vesicles.	Dahhan *et al.* (2022)
	*A. thaliana*	AtFH7	Found in the proteome of clathrin-coated vesicles.	Dahhan *et al.* (2022)
	*A. thaliana*	AtFH10	Found in the proteome of clathrin-coated vesicles.	Dahhan *et al.* (2022)
Other or uncharacterized endomembrane compartments	*A. thaliana*	AtFH3	On endomembranes in pollen tube tips, probably exocytosis related.	Lan *et al.* (2018)
	*A. thaliana*	AtFH4	At mobile compartments adjacent to site of fungal penetration attempt.	Sassman *et al.* (2018)
	*P. patens*	For1A	At endomembrane compartments, probably exocytosis related.	van Gisbergen *et al*. (2020)
	*P. patens*	For1D	At endomembrane compartments, probably exocytosis related.	van Gisbergen *et al*. (2020)

See text for details. PM, plasma membrane; ER, endoplasmic reticulum.

**Fig. 4.**
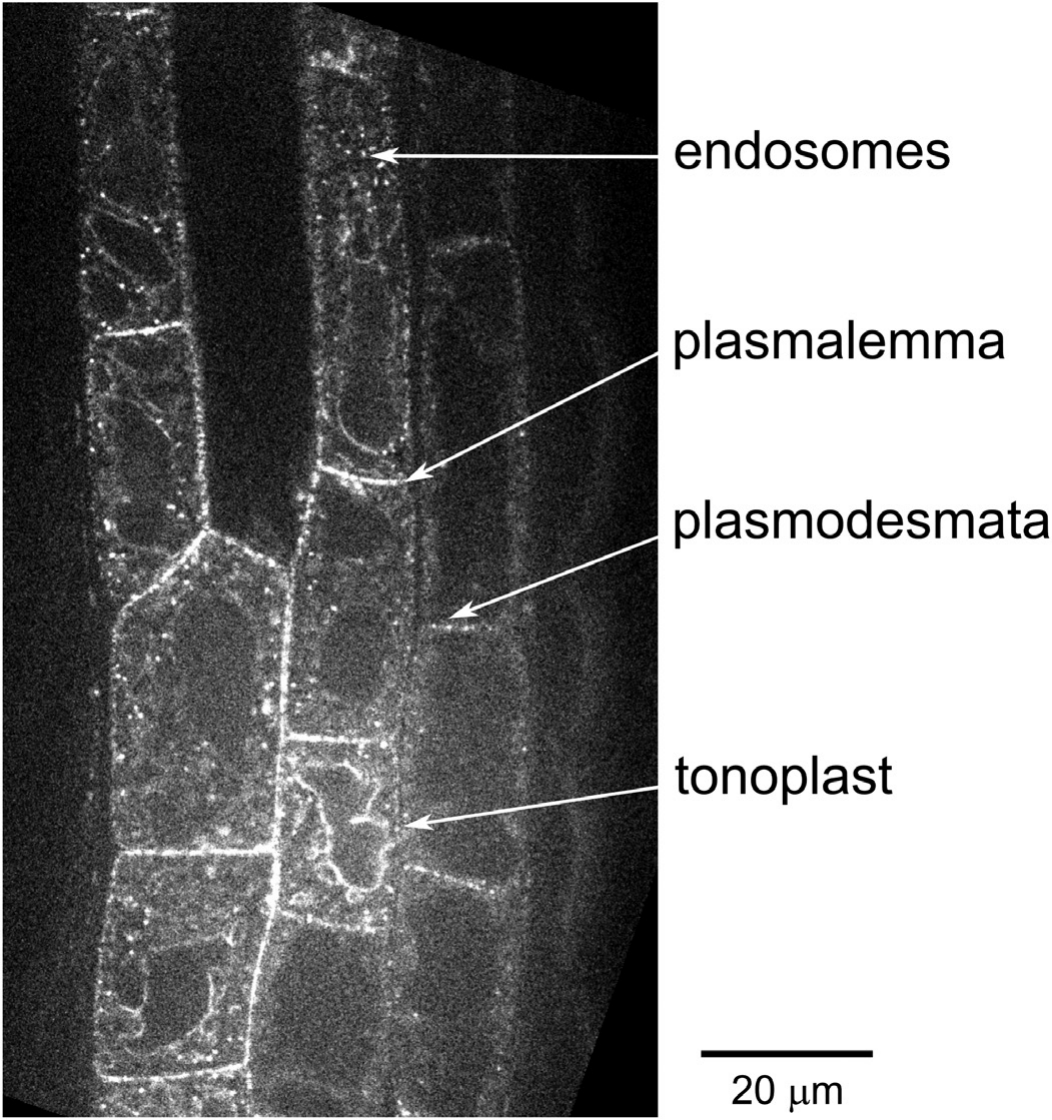
Endomembrane localization of Arabidopsis class I formin AtFH1. A spinning disc confocal microscopy section of the primary root elongation zone rhizodermis from a seedling expressing biologically active GFP-tagged AtFH1 in the genetic background of the *fh1:CRISPR* loss-of-function mutant (Cifrová *et al*., 2020) shows an overlap of several localization patterns found along the developmental gradient of the root tip. For expression construct description, transformation procedure, culture conditions, and imaging methods, see Oulehlová *et al*. (2019).

In Arabidopsis rhizodermis, where the progress of cell differentiation reflects cell position along the root, allowing estimation of the sequence of developmental events from simultaneous observation of distinct root zones, AtFH1 undergoes a series of localization changes. While the protein is predominantly found at the plasmalemma, plasmodesmata, and endosomes in the meristematic zone, in some cells it decorates the tonoplast at or around the time of the vacuolar system rearrangements that give rise to the central vacuole, and subsequently disappears in mature root tissues (Oulehlová *et al.*, 2019; Fig. 4). It remains to be seen whether the tonoplast-localized formin contributes to the developmental dynamics of the vacuole, or whether the tonoplast localization results from processes leading to AtFH1 degradation. While not directly related to membrane trafficking as such, another class I formin, rice OsFH13/DRT, whose mutation causes a pleiotropic cell growth defect resulting in dwarf plant stature and reduced tillering (Ren *et al.*, 2022), also migrates between cellular membranes, relocating from the plasmalemma to the chloroplast envelope in mesophyll cell protoplasts in response to a blue light stimulus. This movement depends on physical interaction between the formin’s FH1 domain and the LOV2 domain of the blue light receptor phototropin, which was documented by yeast two-hybrid assays, as well as by split luciferase assays and co-immunoprecipitation (Y. Zhang *et al.*, 2023).

Endomembrane-related formin roles may be uncovered by the localization and/or phenotypic effects of altered formin proteins, or by consequences of (tagged) formin overexpression. An N-terminally green fluorescent protein (GFP)-tagged version of Arabidopsis AtFH5, presumably incapable of co-translational membrane insertion, decorated the nuclear envelope in the *Nicotiana benthmiana* leaf epidermis expression system, probably due to as yet uncharacterized protein–protein interactions (Cvrčková *et al.*, 2014). In the same heterologous expression system, overexpression of C-terminally GFP-tagged AtFH4 induced co-alignment of the endoplasmic reticulum and cortical microtubules, while a cytoplasm-located AtFH4 derivative did not show this effect (Deeks *et al*., 2010).

Last, but not least, an important line of evidence linking formins to endomembrane trafficking comes from the proteomic characterization of distinct endomembrane compartments. While earlier systematic analyses of various Arabidopsis endomembranes did not reveal any formins associated with specific membrane fractions (Dunkley *et al.*, 2006; Groen *et al.*, 2014; Heard *et al*., 2015), the class I formins AtFH4, AtFH6, AtFH7, and AtFH10 were found on isolated clathrin-coated vesicles in a recent study using more sensitive techniques (Dahhan *et al.*, 2022). Remarkably, no class II formin was identified in the same analysis, indicating a possible specific role for class I formins in clathrin-mediated endocytosis.

## The active cargo model of class I formin function

**Fig. 5.**
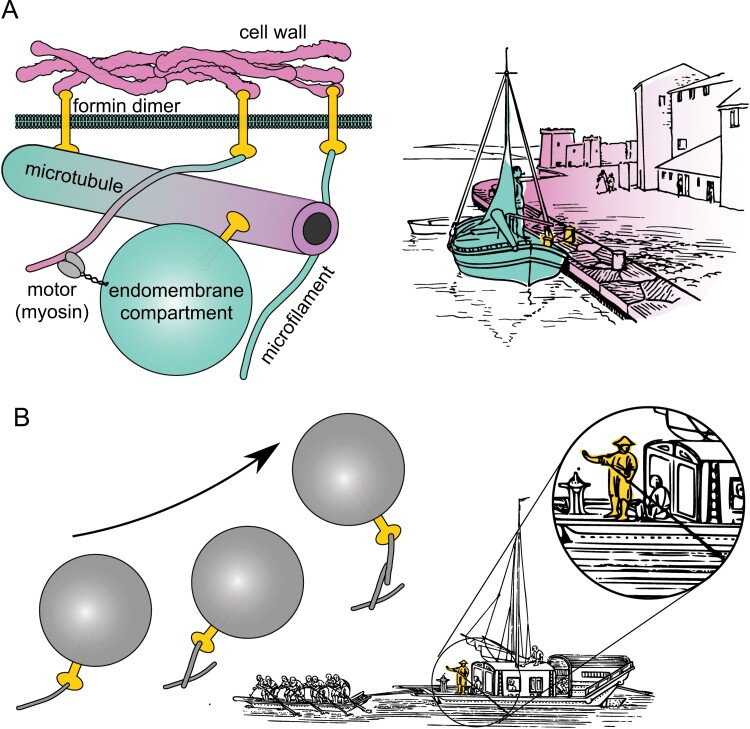
Proposed models of the role of class I formins in endomembrane dynamics. (A) The dock–ship model. (B) The active cargo hypothesis—an example of a non-processive vesicle-borne formin that steers its compartment’s movement by nucleating short actin filaments. The figure includes an anonymous drawing of a docked boat from Wikimedia Commons (https://commons.wikimedia.org/wiki/File:PSF_Q-740001.png; public domain), and an image of a towed Chinese ship by John Gray, 1878; both drawings are from OpenClipart.org and licenced under the Creative Commons Zero 1.0.

With their ability to physically link the cytoskeletal meshwork with extra-cytoplasmic cell wall structures, transmembrane class I formins can mediate ‘docking’ of the dynamic cortical cytoskeleton and its stabilization at specific locations on the cell surface. The cortically anchored cytoskeleton could, at the same time, itself act as a ‘dock’ for relatively more mobile endomembrane compartments that carry transmembrane formins, which, in their turn, may organize cytoskeletal structures around existing endomembrane compartments. Thus, in our ‘ship–dock model’ (first proposed in Cvrčková *et al.*, 2014), the ‘ship’ and ‘dock’ continuously exchange their roles depending on their relative mobility (Fig. 5A).

The above-described model assumes (near-)continuous anchoring of the formin to the cytoskeletal structures. While this may be the case at least for some formin–microtubule interactions, it may not generally hold for formin-mediated actin nucleation. Some, if not all, class I formins are non-processive, as shown for AtFH1 (Michelot *et al*., 2006), that is they detach from microfilament ends after nucleation. Therefore, their role in endomembrane trafficking may be more adequately described as that of ‘active cargoes’, namely proteins that execute (some of) their functions while on their way towards their cellular destination, and, as a result, affect the fate of membrane compartments carrying them, without directly providing mechanical energy for the transport itself. In the case of endomembrane-localized class I formins, this may mean, for example, nucleating microfilaments whose subsequent function does not depend on the formins’ continuous presence (Fig. 5B). Structures assembled from such microfilaments may modulate the movement of vesicles transported directly by motor proteins or by (motor-dependent) bulk cytoplasmic streaming (Lu and Gelfand, 2023).

Can such an ‘active cargo’ model be extended to other situations, involving perhaps not only class I formins, but also other integral membrane proteins? Unless a particular modification at the destination (such as, for example, phosphorylation) is necessary to activate a newly synthesized membrane protein, or unless such a protein is actively maintained in an inactive form until it reaches its destination, a transmembrane protein is likely to exert its function while on its way in the secretory or endocytotic pathway. This, however, does not automatically mean that such a function is biologically relevant, and—even more importantly—whether it affects the trafficking of compartments carrying the protein in question. For example, loading of auxin into exocytotic vesicles by vesicle-borne PIN family auxin carriers could result in auxin release to the periplasm (Baluška *et al.*, 2003), although quantitative modelling reveals that the amount of auxin thus released is unlikely to be sufficient for biologically meaningful effects (Hille *et al.*, 2018). Even if it were sufficient, a mere transporter activity of vesicle-borne PINs would not make them ‘active cargoes’ in our sense, as they would not be modulating the fate of compartments carrying them.

Nevertheless, there might be other transmembrane proteins acting as biologically relevant active cargoes of membrane trafficking. One such candidate is the protein kinase FERONIA (FER), a member of a family of receptor kinases that respond to a variety of peptide or pectin ligands and engage in diverse signalling pathways participating in fertilization, biotic and abiotic stress response, or phytohormone-mediated regulatory processes (see Wang *et al*., 2022). Similar to the formins, FER can modulate nanodomain organization in membranes (Smokvarska *et al.*, 2023). Plasmalemma-localized FER undergoes endocytic recycling via both clathrin-dependent and clathrin-independent pathways. Binding of the RALF1 peptide ligand to FER stimulates clathrin-dependent re-distribution of fluorescent protein-tagged FER from the plasmalemma to endomembrane compartments (Yu *et al*., 2020), while another ligand, the flagellin-derived elicitor peptide flg22, inhibits endocytotic recycling of FER and causes reorganization of FER-containing nanodomains in the plasmalemma (Xing *et al.*, 2022). Thus, the fate of FER-carrying endosomes, as well as of plasma membrane-localized FER, depends on FER–ligand interactions, probably reflecting FER-mediated signalling; that is, FER modulates its own trafficking via a non-motor activity, as expected for an active cargo protein. Thus, generalization of the proposed ‘active cargo’ model to other transmembrane proteins besides formins may be worth considering.

## Concluding remarks

Despite many new findings in almost a decade since the last reviews covering the interplay between plant formin function and membrane trafficking (Cvrčková, 2013; van Gisbergen and Bezanilla, 2013; Cvrčková *et al*., 2014), many old questions remain open and new ones are continuously emerging. What follows is a (necessarily incomplete) list of topics that deserve to be addressed in the near future.

The proposed ‘division of labour’ between class I and class II formins in tip growth should be investigated. The question of whether the model proposed for moss protonemata, where class I formins play a predominantly exocytotic role while their class II counterparts engage mainly in endocytosis, also holds for pollen tubes or root hairs presents an exciting research programme.

The role of class I formins in endomembrane dynamics, especially in central vacuole biogenesis, as well as the biological implications of the presence of some class I formins on clathrin-coated vesicles, suggesting participation of these formins in endocytosis, deserves further attention.

Last, but not least, the ‘active cargo’ model proposed here for the action of class I formins in the endomembrane system may be worth examining as a more general framework for understanding the function of other integral membrane protein families.

## Data Availability

This review contains no new experimental data.

## Abbreviations

DRF: Diaphanous-related formin
FH1: formin homology domain 1
FH2: formin homology domain 2
GBD/FH3: GTPase-binding domain/formin homology domain 3
PAMP: pathogen-associated molecular pattern
PtdIns(3,5)P_2_: phosphoinositide-3,5-bisphosphate
SMIFH2: small molecule inhibitor of FH2.
